# Cardiorenal syndrome and diabetes: an evil pairing

**DOI:** 10.3389/fcvm.2023.1185707

**Published:** 2023-05-10

**Authors:** Ana Belén Méndez Fernández, Ander Vergara Arana, Aleix Olivella San Emeterio, Maria Antonieta Azancot Rivero, Toni Soriano Colome, Maria Jose Soler Romeo

**Affiliations:** ^1^ Department of Cardiology, Hospital Universitario Vall d´Hebron, Barcelona, Spain; ^2^ Department of Nephrology, Hospital Universitario Vall d´Hebron, Barcelona, Spain

**Keywords:** chronic kidney disease, cardiorenal syndrome, heart failure, diabetes mellitus, cardiorenal units

## Abstract

Cardiorenal syndrome (CRS) is a pathology where the heart and kidney are involved, and the deterioration of one of them leads to the malfunction of the other. Diabetes mellitus (DM) carries a higher risk of HF and a worse prognosis. Furthermore, almost half of people with DM will have chronic kidney disease (CKD), which means that DM is the main cause of kidney failure. The triad of cardiorenal syndrome and diabetes is known to be associated with increased risk of hospitalization and mortality. Cardiorenal units, with a multidisciplinary team (cardiologist, nephrologist, nursing), multiple tools for diagnosis, as well as new treatments that help to better control cardio-renal-metabolic patients, offer holistic management of patients with CRS. In recent years, the appearance of drugs such as sodium-glucose cotransporter type 2 inhibitors, have shown cardiovascular benefits, initially in patients with type 2 DM and later in CKD and heart failure with and without DM2, offering a new therapeutic opportunity, especially for cardiorenal patients. In addition, glucagon-like peptide-1 receptor agonists have shown CV benefits in patients with DM and CV disease in addition to a reduced risk of CKD progression.

## 1. Introduction

Heart failure (HF) is a chronic and complex cardiovascular disease, with a prevalence of approximately 2% of adults in industrialized countries (1). As it is a disease that mainly affects the elderly population, this reaches up to 9% of adults over 80 years of age (2). The triad of the cardiorenal syndrome and diabetes mellitus (DM) is common. The proportion of patients with HF who have DM can reach up to 40%, and practically half of the patients with HF also have chronic kidney disease (CKD) (3).

Patients with diabetes have an increased risk of suffering from HF, and the coexistence of both confers a worse prognosis for patients with HF compared to those without DM (4). In the Framingham cohort, the risk of HF was greater in the presence of DM, especially in women where the risk increased 4-fold, even adjusting for other cardiovascular risk factors (5). The presence of HF and DM is associated with a higher proportion of ischemic etiology, in addition to obesity, hypertension, kidney disease, and even higher natriuretic peptide values than non-DM patients, which could be related to a worse prognosis for these patients (6).

Almost half of the people with DM will have CKD, for that reason DM is the main cause of kidney failure (3). As expected, the combination of the triad of cardiorenal syndrome and diabetes is associated with increased risk of hospitalization and mortality. The presence of shared cardiovascular risk factors, as well as the well-known neurohormonal activation, inflammation, and oxidative stress, may be part of the pathophysiology that explains this complex triad. The complexity of these patients warrants a multidisciplinary approach (4).

Hence, we propose a comprehensive review of the new aspects in cardiorenal syndrome (CRS) in the setting of diabetes, focusing on new and classical protective mechanisms of diabetic nephropathy, biomarkers and reviewing the new therapies for CRS in patients with DM.

## 2. New aspects in cardiorenal syndrome

Cardiorenal syndrome (CRS) is a pathology where the heart and kidney are involved, and the deterioration of one leads to the malfunction of the other. Although its first appearance in the literature was in 1836 (7), it was not until 2008 when its current definition came into force, defining 5 groups, which differ depending on whether the first organ affected is the heart or the kidney, and whether its onset is due to an acute or chronic pathology, reserving a fifth group for a pool of patients with systemic pathologies (8). The five subtypes are outlined in Table 1.

**Table 1:** Classification of cardiorenal syndromes.

CRS type	Nomenclature	Physiopathology	Example
1	Acute CRS	Acute HF resulting in AKI	Renal hypoperfusion by cardiogenic shock
2	Chronic CRS	Chronic HF resulting in CKD	Renal damage by chronic HF
3	Acute RCS	AKI resulting in Acute HF	HF by volume overload in patients with severe AKI
4	Chronic RCS	CKD resulting in Chronic HF	Chronic HF secondary to LVH in patients with CKD-associated cardiomyopathy
5	Secondary CRS	Systemic disease resulting in HF and kidney disease	Lupus, vasculitis, diabetes, sepsis, or amyloidosis

CRS, cardiorenal syndrome; RCS, renocardiac syndrome; HF, heart failure; AKI, acute kidney injury; CKD, chronic kidney disease; LVH, left ventricular hypertrophy.

Although CRS definition and its subtypes has not changed, several authors have been published about its pathophysiology, diagnostic tools—including biomarkers and imaging tests—as well as pharmacological treatment and the need for a multidisciplinary approach through cardiorenal units (CRU) (9, 10).

Patients with CRS and DM are more complex patients, and generally more comorbid than those without DM (11). Management of these patients in CRU allows a better understanding of its epidemiology, pathophysiology, phenotypes, diagnostic tools, and medical treatment (12). The creation of CRU is endorsed by scientific societies with the aim of being able to offer individualized treatment efficiently and safely (13). Close work between cardiologists and nephrologists is essential to properly assess congestion, using biomarkers and imaging tests, and deciding the best treatment in each moment, from diuretics in a decompensation, to prognostic treatment in HF, as well as propose the type and appropriate time of starting renal replacement therapies when indicated.

## 3. Diabetes and heart failure

Diabetes mellitus is a global pandemic with increasing prevalence up to 8.5% of the global adult population from the USA in 2014, most of the individuals being type 2 diabetes (T2DM) (14, 15). Prevalence of T2DM among HF patients shows wide regional and ethnic variations. For example, in a prospective study of acute HF patients admitted to a hospital in Denmark, where 72.4% were males and all Caucasic, 25% of patients were found to have T2DM (14) Another study comparing a southeast Asian cohort from Singapur with a Swedish cohort, 57% of Asians in Southeast Asia were T2DM compared to 24% of white patients from Sweden, despite younger age and less obesity in the former group, with an adjusted odds of diabetes 3.1 times higher in the multivariant analysis (16). In the Reykjavík study (17), a population-based cohort study during 1967–1997 followed until 2002, the prevalence of HF was 11.8% among those with DM2, compared to 3.2% in non-DM patients. Observational studies have consistently demonstrated a 2- to 4-fold increased risk of HF in individuals with DM compared with those without DM (15). Furthermore, prevalence might be even higher for hospitalized patients, around 40%–45% (14, 18). Nevertheless, a better definition, especially of HF with preserved ejection fraction (HFpEF), which is complex in many cases, would help to refine the precision of these estimates (19).

It has been robustly shown that T2DM is associated with worse clinical outcomes and increased mortality, compared to HF patients without T2DM (14). A meta-analysis including 381,725 HF patients showed a higher risk of all-cause death and CV death for patients with T2DM (20). In such a close relationship between T2DM and HF, sodium-glucose cotransporter 2 inhibitors (SGLT2i) have proven to be a powerful tool for the treatment of both pathologies, to the point that the latest AHA/ACC/HFSA guidelines recommend their use in stage A HF (21).

**Figure 1:**
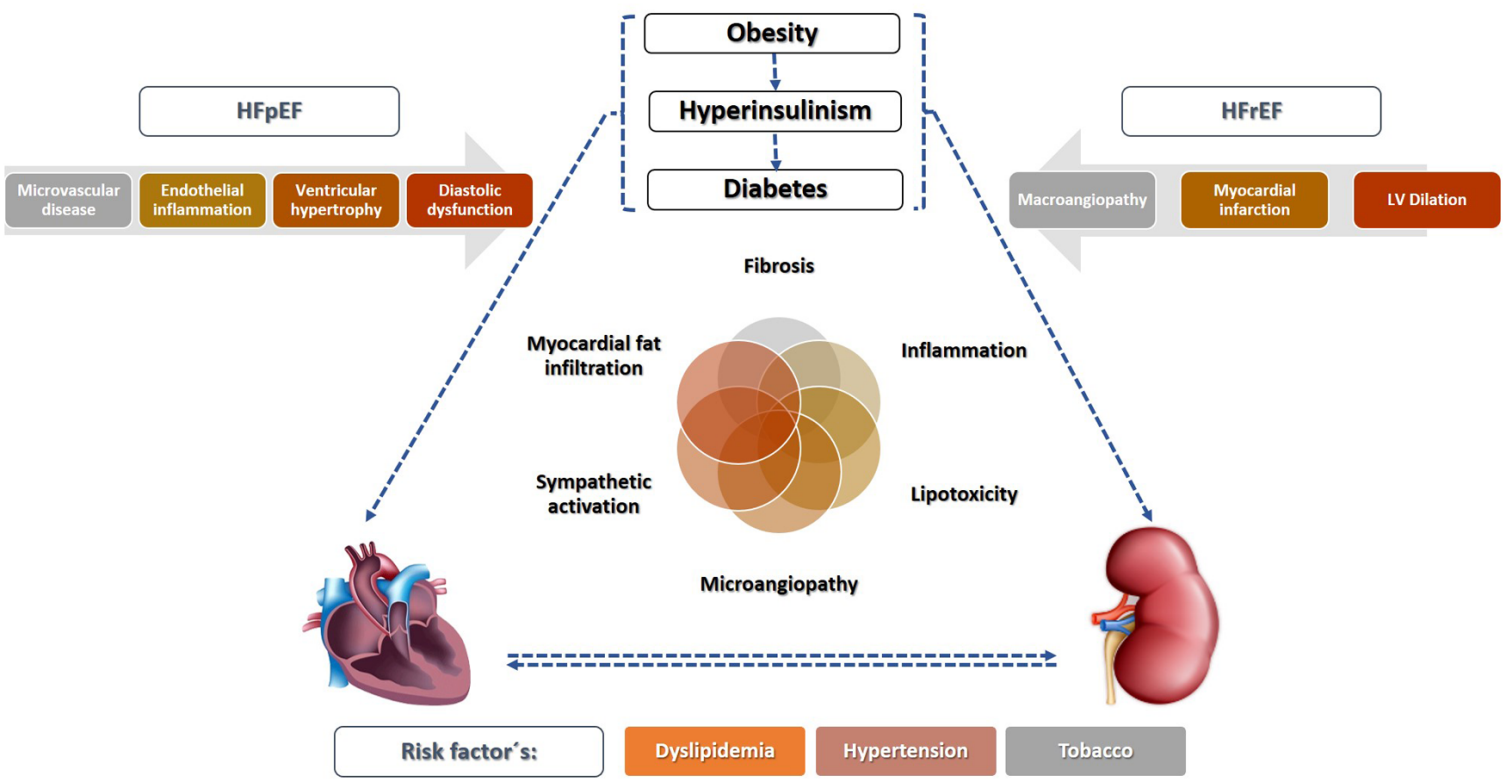
Mechanisms responsible for diabetic cardiorenal injury. Type 2 diabetes, usually linked to obesity and insulin resistance, is sufficient to activate proinflammatory pathways both in the tissue and endothelial cells that contribute tissue injury and fibrosis in the heart (diabetic cardiomyopathy) and the kidney (diabetic nephropathy). In addition, risk factors linked to diabetes such as dyslipidemia or hypertension also contribute to cardiorenal injury. HFpEF, heart failure with preserved ejection fraction; HFrEF, heart failure with reduced ejection fraction.

Classically, the higher prevalence of HF in diabetes was explained by coexisting comorbidities that cause HF, such as hypertension and coronary artery disease (CAD) (Figure 1), but recently the direct detrimental effect of T2DM on the myocardium has appeared as a key feature (22, 23). In contrast, type 1 diabetes mellitus (T1DM) does not seem to trigger the development of HF “*per se*”, but through hypertension and CAD (24), and it has been demonstrated that animal models of T1DM do not present fibrosis, hypertrophy, inflammation, or oxidative stress (23).

What is known as Diabetic Cardiomyopathy is defined as the presence of a structurally and functionally abnormal myocardium in the absence of a heart disease that justifies it in patients with DM (22). Two distinct phenotypes can be drawn based on ejection fraction, although this approach might be oversimplistic: HFpEF, linked to left ventricular diastolic dysfunction and hypertrophy (25) and HF with reduced ejection fraction (HFrEF), considered to be caused by CAD, usually diffuse, multi-vessel, and sometimes with silent myocardial infarction (22). Nevertheless, accumulating evidence points towards an independent effect mainly directed by hyperinsulinaemia, even before T2DM is diagnosed (14). A complex interrelation of several pathways is involved, causing the deposition of advanced glycosylation end-products, lipotoxicity and microvascular rarefaction (22, 26, 27) leading to several maladaptive responses resulting in myocyte damage. A review from Milton Packer (23), discusses the role of hyperinsulinaemia as a central key feature of how T2DM promotes HF by suppression of autophagy and promotion of oxidative stress and mitochondrial dysfunction, causing myocyte damage, and at the same time, activating sodium-hydrogen exchangers in cardiomyocytes and in the proximal renal tubules (sodium retention and increased filling pressures). Also, epicardial adipose tissue expansion has been consistently reported in DM, resulting in secretion of proinflammatory adipocytokines acting as a paracrine organ on the myocardium, and causing microvascular dysfunction, inflammation, and fibrosis (28, 29). The predominance of cardiomyocyte death would lead towards a HFrEF phenotype, and in contrast, HFpEF would emerge in cases with prominent coronary microvascular endothelial inflammation, with concentric left ventricle remodeling and higher stiffness (22, 30).

## 4. New and classical protective mechanisms of diabetic nephropathy

**Figure 2:**
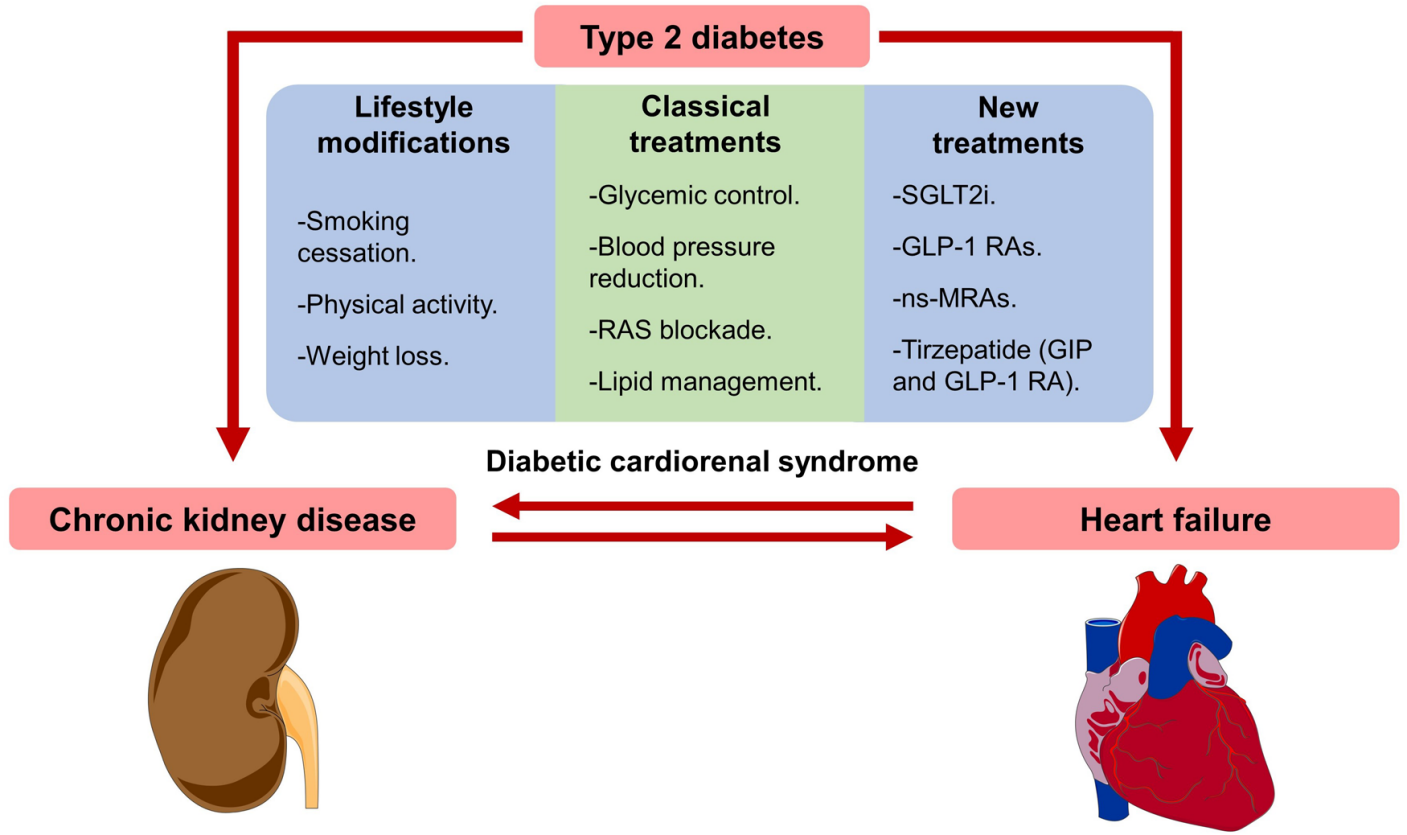
Diabetic cardiorenal injury and preventive treatments. Type 2 diabetes contributes to both diabetic cardiomyopathy and nephropathy. Moreover, heart failure can produce chronic kidney disease, and kidney injury can cause heart failure. Treatments to prevent diabetic cardiorenal injury include lifestyle modifications, glucose-lowering therapies, lipid management (usually with statins), and renin-angiotensin system (RAS) blockade. Sodium-glucose cotransporter 2 inhibitors (SGLT2i) and glucagon-like peptide 1 receptor agonists (GLP-1 RAs) have now become first-line glucose-lowering therapies along with metformin. In addition, non-steroidal mineralocorticoid receptor antagonists (ns-MRAs) and Tirzepatide (glucose-dependent insulinotropic polypeptide and GLP-1 receptor agonist) will soon be part of the new standard of care. The Figure was partly generated using Servier Medical Art (31).

Before 2016, the treatment of patients with T2DM and CKD has been focused on four main interventions: (A) reduce cardiovascular risk factors, (B) hyperglycemia treatment, (C) control blood pressure (BP), and (D) initiate renin-angiotensin system inhibitors (RASi) (Figure 2). Among the modifiable risk factors, smoking cessation and weight loss linked to dietary changes are probably the two most important, although well-designed studies evaluating the impact of these changes are scarce (32).

Blood glucose control, avoiding the risk of hypoglycemia, proved to be more effective in preventing CV events than renal events. Intensive glucose-lowering therapy reduced microvascular complications and albuminuria development in T1DM diabetic patients (33). However, in T2DM patients, results were more controversial with the intensive control even related to an increased CV and all-cause mortality in the ACCORD (*Action to Control Cardiovascular Risk in Diabetes*) trial related to a higher risk of hypoglycemia (34) and little or no prevention of renal events (34, 35). In addition, the *United Kingdom Prospective Diabetes Study* (UKPDS) demonstrated that an HbA1c target of 7% could be sufficient to prevent CV events, minimizing the risk of hypoglycaemia, but without offering any additional renal protection (36). Of note that these two studies were performed before the development of the new drugs with few severe hypoglycaemia adverse events such as SGLT2is and glucagon-like peptide-1 receptor agonists (GLP-1 RAs).

BP reduction below 140/90 mmHg, regardless of the drug classes employed, has been shown to decrease mortality and CV events in DM patients (37). Moreover, in patients with DM and CKD (DKD), BP control is more effective in preventing albuminuria but without any effect on creatinine clearance or CKD progression in T2DM patients unless RASi is used as antihypertensive therapy (37, 38). Specific inhibition of RAS demonstrated a reduction of CV and renal events in both T1DM and T2DM patients (39, 40). Regarding renal outcomes, the Collaborative Study Group was the first to showed ACE inhibitor-mediated renal protection in T1DM patients (41). Patients were randomized to captopril 25 mg three times daily versus placebo, and after 3 years of follow-up, the captopril group showed a 48% reduction in the risk of doubling of serum creatinine and a 50% reduction in the combined endpoint of death, need for dialysis, or kidney transplantation. In the same way, later studies with angiotensin receptor blockers (ARBs) also proved to prevent CKD progression in T2DM patients. The IDNT (*Irbesartan Diabetic Nephropathy Trial*) with 300 mg of irbesartan, 10 mg of amlodipine, or placebo, shown that Irbesartan was superior to both placebo and amlodipine in reducing the risk of doubling of serum creatinine, end-stage kidney disease (ESKD), or all-cause mortality (42). Additionally, the RENAAL (*Reduction in End-Points in Non-Insulin Dependent Diabetes Mellitus With the Angiotensin II Antagonist Losartan*) trial assigned T2DM patients to treatment with losartan versus placebo, demonstrating a 16% risk reduction in the losartan group in the risk of doubling serum creatinine, CKD, and all-cause mortality (40).

Considering the beneficial effects of both ACEi and ARB, the current 2022 KDIGO (*Kidney Disease Improving Global Outcomes*) guidelines recommend the use of RASi in patients with CKD and DM in the presence of albuminuria, regardless of whether there is hypertension or not (43). Studies like INNOVATION (*Incipient to Overt: Angiotensin II Blocker, Telmisartan, Investigation on Type 2 Diabetic Nephropathy*), where Telmisartan reduced the progression to overt albuminuria being 30.9% of the included patients normotensive (44), support the latter suggestion. Nevertheless, when initiating RAS blockade in normotensive patients, BP should be closely monitored because pressures below 120 mmHg and hypotensive episodes have also been related to increased mortality, as shown by studies like IDNT (45). Moreover, the combination of ACEi and ARB should be avoided after the ONTARGET (*Ongoing Telmisartan Alone and in Combination With Ramipril Global Endpoint*) and VA NEPHRON-D (*Veterans Affairs Nephropathy in Diabetes*) did not show cardiorenal benefit and did show an increased risk of adverse renal events (hyperkalemia, acute kidney injury, or urgent need for dialysis) (46).

In recent years, another molecule, a combination of neprilysin inhibitor and ARB, has presented data suggesting an additional nephroprotective effect, relative to ACE inhibitors. A subanalysis of PARADIGM-HF (47) found a slower rate of decline in iGFR and better cardiovascular outcomes, even in patients with chronic kidney disease. In another similar sub-analysis in patients with chronic HF and DM2, the addition of the combination of neprilysin inhibitor + ARBs slowed the deterioration of renal function (48).

After 2016, the cardiorenal protective effects demonstrated by two promising glucose-lowering drug classes, the SGLT2is and GLP-1 RAs, have recently changed the treatment of CV and renal complications of diabetes (49, 50) (Figure 2).

## 5. New therapies in cardiorenal syndrome in diabetes

Patients with DM2 and CKD have a high burden of cardiovascular disease and a very high risk of kidney disease progression, even in patients with optimal management. With the need to improve the cardiovascular profile of these patients, new classes of antidiabetic agents, including SGLT2is and GLP-1 RAs, have shown a significant reduction in cardiovascular and renal outcomes in patients with T2DM and CKD. These drugs have revolutionized the management of patients with diabetic CKD with high risk for cardiovascular events, independent of glycemic control.

### 5.1. Cardiovascular and renal outcomes with inhibitors of SGLT2

The *United States Food and Drug Administration* (FDA) proposed to evaluate all new drugs for glycemic control, in addition to their efficacy as a treatment for hyperglycemia in DM, as well as their cardiovascular safety (51). This approach was motivated, in part by the high prevalence of cardiovascular disease in DM, and concerns about a poor cardiovascular benefit with different antidiabetic drugs, such as rosiglitazone (52), pioglitazone, peroxisome proliferator–activated receptor (PPAR) agonist muraglitazar (53) and sulphonylureas (54).

In randomized clinical trials (RCT) involving patients with T2DM the term MACE (*Major Adverse Cardiovascular Event*) has been defined as a primary cardiovascular adverse effect (55), and this cardiovascular endpoint include death from a cardiovascular cause, nonfatal myocardial infarction, and nonfatal stroke. Some studies have also included hospitalization for unstable angina and/or added hospitalization for HF. In this regard, RCT with SGLT2is in diabetics such as EMPA-REG (*Empagliflozin Cardiovascular Outcome Event Trial Type 2 Diabetes Mellitus Patients–Removing Excess Glucose*) (50) and CANVAS trial (*Canagliflozin Cardiovascular Assessment Study*) (56), have demonstrated cardiovascular benefits in reduction of MACE and risk of hospitalization associated with HF. Subsequently, VERTIS CV (*Cardiovascular Outcomes Following Ertugliflozin Treatment in Type 2 Diabetes Mellitus Participants With Vascular Disease*) (57) and DECLARE-TIMI 58 (*Dapagliflozin Effect on Cardiovascular Events–Thrombolysis in Myocardial 58*) (58) failed to demonstrate reduction of MACE, but they observed reduction in risk for HF. Insight of this outcomes, trials such as DAPA-HF (*Dapagliflozin in Patients With Heart Failure and Reduced Ejection Fraction*) (59), EMPEROR-Reduced (*Cardiovascular and Renal Outcomes with Empagliflozin in Heart Failure Reduced Ejection Fraction*) (60), DELIVER (*Dapagliflozin in Heart Failure with Mildly Reduced or Preserved Ejection Fraction*) (61) and EMPEROR-preserved (*Empagliflozin in Heart Failure with a Preserved Ejection Fraction*) (62) were designed with the primary outcome of risk of hospitalization for HF and cardiovascular death. All showed an important reduction of the risk for HF, regardless the absence or presence of T2DM. This benefit in patients with stable HF was also found in patients with recent decompensate HF and T2DM. SOLOIST-WHF (*Sotagliflozin in Patients with Diabetes and Recent Worsening Heart Failure*) evidenced that sotagliflozin initiated before or shortly after discharged was associated with reduce risk for mortality from cardiovascular deaths and urgent visits for HF in comparison with placebo (62). In fact, the use of dapagliflozin and empagliflozin are strongly recommended in patients with HFrEF according to current guidelines for the diagnosis and treatment of HF, and recent studies have also shown their benefit in HFpEF (61, 62).

In terms of renal benefits, MARE (*Major Adverse Renal Events*) has been used to define renal outcomes in RCT in diabetes. The vast majority of the antidiabetic drugs studies consider renal outcomes as a secondary endpoint. These outcomes are defined as (A) incident renal disease (onset of sustained eGFR measurable kidney injury <60 ml/min/1.73 m^2^ and/or onset of albuminuria (UACR > 30 mg/g), (B) worsening kidney disease (sustained > 40% reduction in GFR and/or significant increase in UACR), (C) need to start renal replacement therapy (RRT), and (D) renal death (death directly attributable to kidney disease) (63). Recently, several RCT such as CREDENCE (*Canagliflozin and Renal Events in Diabetes With Established Nephropathy Clinical Evaluation*) (64), DAPA-CKD (*Dapagliflozin in Patients with Chronic Kidney Disease*) (65) and EMPA-KIDNEY (*Empagliflozin in Patients with Chronic Kidney Disease*) (66) have evaluated the renal outcome as a primary endpoint, and have observed an important reduction of MARE in the treatment arm groups. Table 2 summarizes the baseline characteristics of different trials and Table 3 the main results of SGLT2i driven cardiorenal protection.

**Table 2:** Baseline study and patient characteristics in the cardiovascular and renal outcomes trials of SGLT2is.

Trial	Drug	Dose (mg)	*N*	Diabetes (%)	HFpEF (%)	HFrEF (%)	Basal renal characteristics
EMPA-REG	Empagliflozin	2,510	7,020	100	NA	NA	25.9% with eGFR <60 ml/min/1.73 m^2^
CANVAS	Canagliflozin	300,100	10,142	100	NA	NA	20.1% with eGFR <60 ml/min/1.73 m^2^
DECLARE-TIMI 58	Dapagliflozin	10	17,160	100	NA	NA	7.1% with eGFR <60 ml/min/1.73 m^2^
VERTIS CV	Ertugliflozin	515	8,246	100	NA	NA	21.8% with eGFR <60 ml/min/1.73 m^2^
DAPA HF	Dapagliflozin	10	4,744	41.8	–	100	All patients had eGFR >30 ml/min/1.73 m^2^
EMPEROR REDUCE	Empagliflozin	10	3,730	49.8	–	100	48% with eGFR <60 ml/min/1.73 m^2^
EMPEROR PRESERVE	Empagliflozin	10	5,988	49	100	–	50% with eGFR <60 ml/min/1.73 m^2^
SOLOIST-WHF	Sotagliflozin	NA	1,222	100	40.5	59.5	All patients had eGFR >30 ml/min/1.73 m^2^
DELIVER	Dapagliflozin	10	6,263	44.7	65.9	Mildly reduced EF: 34.1	All patients had eGFR >25 ml/min/1.73 m^2^
CREDENCE	Canagliflozin	100	4,401	100	NA	NA	eGFR <30–89 ml/min/1.73 m^2^ and UACR 300–500 mg/g
DAPA-CKD	Dapagliflozin	10	4,304	67.6	NA	NA	45% with eGFR 30–44 ml/min/1.73 m^2^ and 67% with UACR >1,000 mg/g
EMPA-KIDNEY	Empagliflozin	10	6,609	46	NA	NA	44% with eGFR 30–44 ml/min/1.73 m^2^ and 51.8% with UACR >300 mg/g

EMPA-REG OUTCOME, empagliflozin cardiovascular outcome event trial in type 2 diabetes mellitus patients–removing excess glucose; CANVAS, canagliflozin cardiovascular assessment study program; DECLARE-TIMI 58, dapagliflozin effect on cardiovascular events–thrombolysis in myocardial infarction; VERTIS CV, cardiovascular outcomes following ertugliflozin treatment in type 2 diabetes mellitus participants with vascular disease; DAPA-HF, dapagliflozin in patients with heart failure and reduced ejection fraction; EMPEROR-reduced, cardiovascular and renal outcomes with empagliflozin in heart failure; EMPEROR-preserved, empagliflozin in heart failure with a preserved ejection fraction; SOLOIST-WHF, sotagliflozin in patients with diabetes and recent worsening heart failure; DELIVER, dapagliflozin in heart failure with mildly reduced or preserved ejection fraction; CREDENCE, canagliflozin and renal events in diabetes with established nephropathy clinical evaluation; DAPA-CKD, dapagliflozin in patients with chronic kidney disease; EMPA-KIDNEY, empagliflozin in patients with chronic kidney disease; HFpEF, heart failure preserve ejection fraction; HFrEF, heart failure reduce ejection fraction; eGFR, estimated glomerular filtration rate; UACR, urinary albumin-to-creatinine ratio; NA, not available.

**Table 3:** Principal cardiovascular and kidney outcomes reported in cardiovascular outcomes trials with SGTL2is.

Trial	MACE HR (95% CI)	HF hospitalization HR (95% CI)	MARE HR (95% CI)	UACR progression	All-cause mortality HR (95% CI)
EMPA-REG	0.86 (0.74–0.99)	0.62 (0.45–0.86)	Doubling creatinine, eGFR ≤45 ml/min/1.73 m^2^, RRT or death from renal disease: HR 0.54 (0.40–0.75)	0.62 (0.54–0.72)	0.68 (0.57–0.82)
CANVAS	0.86 (0.75–0.97)	0.67 (0.52–0.87)	40% reduction in eGFR, RRT or death from renal disease: HR 0.60 (0.47–0.77)	0.73 (0.67–0.79)	0.87 (0.74–1.01)
DECLARE-TIMI 58	0.93 (0.84–1.03)	0.73 (0.61–0.88)	≥40% reduction in eGFR to <60 ml/min/1.73 m^2^: HR 0.53 (0.43–0.66)	NA	0.93 (0.82–1.04)
VERTIS CV	0.97 (0.85–1.11)	0.70 (0.54–0.90)	Doubling creatinine, RRT or death from renal disease: HR 0.81 (0.63–1.04)	NA	0.93 (0.80–1.08)
DAPA HF	–	0.70 (0.59–0.83)	≥50% reduction in eGFR, RRT: HR 0.71 (0.44–1.16)	NA	0.83 (0.71–0.97)
EMPEROR REDUCE	–	0.70 (0.58–0.85)	Mean slope of change in eGFR (ml/min/1.73 m^2^): −0.55 ± 0.23 vs. −2.28 ± 0.23 HR 1.73 (1.10–2.23) Reduce eGFR and RRT: HR 0.50 (0.32–0.77)	NA	0.92 (0.77–1.10)
EMPEROR PRESERVE	–	0.71 (0.60–0.83)	Mean slope of change in eGFR (ml/min/1.73 m^2^): −1.25 ± 0.11 vs. −2.62 ± 0.11 HR: 1.36 (1.06–1.66) Reduce eGFR and RRT: HR 0.95 (0.73–0.1.24)	NA	1.00 (0.87–1.15)
SOLOIST-WHF	–	0.64 (0.49–0.83)	Change in eGFR (ml/min/1.73 m^2^): −0.34 (sotaglifozin) vs. −0.18 (placebo) HR −0.16 (−1.3 to 0.98)	NA	0.82 (0.59–1.14)
DELIVER	–	0.79 (0.69–0.91)	NA	NA	0.94 (0.93–1.07)
CREDENCE	0.80 (0.67–0.95)	0.69 (0.57–0.83)	Doubling creatinine, RRT or death from renal or cardiovascular disease: HR 0.70 (0.50–0.72)	Geometric mean of UACR was 31% lower in canagliflozin group	0.83 (0.68–1.02)
DAPA-CKD	–	Death from CV causes or hospitalization for HF: 0.71 (0.55–0.92)	Decline of eGFR at least 50%, ESKD and death from renal disease: HR 0.56 (0.45–0.68)	Geometric mean of UACR was 29.3% lower in dapagliflozin group	0.69 (0.53–0.88)
EMPA-KIDNEY	–	Death from CV causes or hospitalization for HF: 0.84 (0.67–0.1.07)	Primary outcome was the first occurrence of progression of kidney disease or death from cardiovascular causes: HR 0.72 (0.64–0.82)		0.87 (0.70–1.08)

EMPA-REG OUTCOME, empagliflozin cardiovascular outcome event trial in type 2 diabetes mellitus patients–removing excess glucose; CANVAS, canagliflozin cardiovascular assessment study program; DECLARE-TIMI 58, dapagliflozin effect on cardiovascular events–thrombolysis in myocardial infarction; VERTIS CV, cardiovascular outcomes following ertugliflozin treatment in type 2 diabetes mellitus participants with vascular disease; DAPA-HF, dapagliflozin in patients with heart failure and reduced ejection fraction; EMPEROR-reduced, cardiovascular and renal outcomes with empagliflozin in heart failure; EMPEROR-preserved, empagliflozin in heart failure with a preserved ejection fraction; SOLOIST-WHF, sotagliflozin in patients with diabetes and recent worsening heart failure; DELIVER, dapagliflozin in heart failure with mildly reduced or preserved ejection fraction; CREDENCE, canagliflozin and renal events in diabetes with established nephropathy clinical evaluation; DAPA-CKD, dapagliflozin in patients with chronic kidney disease; EMPA-KIDNEY, empagliflozin in patients with chronic kidney disease; MACE, major adverse cardiovascular events; HF, heart failure; MARE, major adverse renal events; eGFR, estimated glomerular filtration rate; RRT, renal replacement therapy; UACR, urinary albumin-to-creatinine ratio; NA, not available; CV, cardiovascular.

The mechanisms that explain the effects of SGLT2is on HF outcomes and progression of kidney disease are possibly multifactorial. SGLT2is produce natriuresis, reduce volume overload and have direct hemodynamic effects by decreasing glomerular hyperfiltration (67). Moreover, SGLT2is are associated with reduced inflammation and oxidative stress (68), improved cardiovascular efficiency, blood pressure control, and weight loss, which are factors linked to HF outcomes (69). CRS is a bidirectional relationship between heart and kidney and in presence of diabetes confers an increased risk of mortality and reduced quality of life of patients. Currently, SGLT2is constitute a real option for treatment of CRS in diabetes, because of its cardio and renoprotective effect beyond glycaemic control.

SGLT2is act by inhibiting SGLT2 expressed in proximal convoluted tubule (PCT) lowering glucose reabsorption. This increase of glucose excretion results in better glycemic control. Moreover, in normal condition, Na+ is cotransported with glucose through SGLT2 and when SGLT2 is inhibited, it results in natriuresis and osmotic diuresis. The increase in Na+ that reaches the macula densa facilitates the activation of TGF, which leads to vasoconstriction of the afferent arteriole and therefore a reduction in the glomerular filtration rate, and finally a decrease of glomerular hyperfiltration and prevention of glomerular damage with reduction of progression of CKD. Moreover, SGLT2is interferes with the sodium-hydrogen exchanger (NHE-3), which is responsible for tubular reuptake of Na+ after filtration, and increases markedly in HF, and may be responsible for resistance to diuretics and peptides endogenous natriuretics (70). This is an important finding since it is known that natriuresis play an important role in control of congestion in HF and CRS (71). Another important reno and cardioprotective mechanism is BP control that results in a decrease in afterload. It has been described important reduction in BP in 24 h ambulatory blood pressure monitoring (24 h ABPM), including nocturnal BP with dapagliflozin and empagliflozin (72), which further demonstrates the cardiovascular protective effects of SGLT2is. Taken together these studies, in patients with T2DM at risk for HF and progression of CKD, the use of SGLT2is have to be mandatory. SGLT2i have been demonstrated an enormous impact in management of patients with HF and CRS.

### 5.2. Cardiovascular and renal outcomes with GLP-1 receptor agonists

Cardiovascular benefits associated with GLP-1 are multiple. It is known that inflammation play an important role in atherosclerosis development. GLP-1 in different studies reduce endothelial disfunction and inflammation (73) which can decrease the risk and progression of atherosclerotic plaque. Moreover, it has been described an improvement in lipid metabolism (74) and reduction in blood pressure due to natriuretic effects (75). These mechanisms can act synergistically improving the cardiovascular profile of the diabetic patient. Different RCT have demonstrated the CV benefits in treatment with GLP-1 RAs in patients with diabetes and established CV disease, however the evidence in HF prevention is scarce.

The LEADER trial (Liraglutide and Cardiovascular Outcomes in Type 2 Diabetes) (76), SUSTAIN-6 (Trial to Evaluate Cardiovascular and Other Long-term Outcomes With Semaglutide in Subjects With Type 2 Diabetes) (49), REWIND trial (Dulaglutide and Cardiovascular Outcomes in Type 2 Diabetes) (77) and HARMONY Outcomes (Effects of Albiglutide on Major Cardiovascular Events in Patients With Type 2 Diabetes Mellitus) (78) have demonstrated a reduction of risk for composite cardiovascular outcome defined as nonfatal MI, nonfatal stroke, or cardiovascular death. Although cardiovascular superiority was also demonstrated with albiglutide this therapy has been withdrawn from the market. In the EXSCEL trial (Effects of Once-Weekly Exenatide on Cardiovascular Outcomes in Type 2 Diabetes) including patients with T2DM with or without prior cardiovascular disease, the incidence of MACE did not differ between exenatide and placebo group (79). In the ELIXA trial (Lixisenatide in Patients with Type 2 Diabetes and Acute Coronary Syndrome), the only GLP-1 RA trial conducted in a post–acute coronary syndrome setting, it was observed that the use of lixisenatide was not superior to placebo in reducing the risk of MACE (80). The PIONEER-6 trial (Oral Semaglutide and Cardiovascular Outcomes in Patients With Type 2 Diabetes) demonstrated noninferiority with the oral formulation of semaglutide with respect to the primary composite outcome for major adverse cardiovascular events (81). Oral semaglutide is undergoing further specific cardiovascular outcome testing in the SOUL trial (A Heart Disease Study of Semaglutide in Patients With Type 2 Diabetes; URL: ClinicalTrials.gov. Unique identifier: NCT03914326). Given the cardiovascular benefits, GLP-1 RA are strongly recommended in patients with T2DM with high and very high cardiovascular risk (82).

Most of the GLP-1 RA RCTs included few patients with HF (prevalence between 9% and 24%), and the risk of hospitalization for HF was included as a secondary end point. Globally, GLP-1 RAs had a neutral effect on risk for HF. However, two meta-analysis including 8 GLP-1 RAs RCT found hospitalization for HF to be reduced by 10%–11% (83). It is important to note that HARMONY Outcomes and AMPLITUDE-O trial (*Cardiovascular and Renal Outcomes with Efpeglenatide in Type 2 Diabetes*) (84) were the studies with the most marked risk reduction in HF.

To date, there are not RCT that evaluate the efficacy of GLP-1 RA in reduce risk of hospitalization for HF. However, there are three small RCT that evaluate the role of GLP-1 RAs on hospitalization for HF and ventricular function in HFrEF patients. The LIVE trial (*Effect of Liraglutide on Left Ventricular Function in Stable Chronic Heart Failure Patients*) randomized 241 patients with HFrEF with or without T2DM to placebo or liraglutide for 24 weeks. No changes in ventricular function, quality of life, or functional class were observed. But, an increase in heart rate and serious adverse cardiac events occurred more often with liraglutide group (85). The FIGHT trial (*Functional Impact of GLP-1 for HF Treatment*) included 300 HFrEF patients with or without T2DM with a recent decompensation and were randomized to liraglutide or placebo for 6 months (86). The trial showed no significant differences in terms of HF-related events or functional capacity. Another smaller study evaluated albiglutide 30 mg versus placebo in patients with HFrEF and DM, finding no differences in LVEF, the 6-min test, or myocardial metabolism (87).

Therefore, there is insufficient evidence on the benefit of GLP-1 RAs in patients with HF. Certainly, it is important to identify patients at risk of HF and patients diagnosed with HF, especially those with HFrEF due to possible cardiac adverse events. There are no data available in patients with HFpEF, but more than 80% of these patients are overweight or obese, therefore, taking into account that GLP-1 RAs produce a weight loss that varies between 5% and 10%, they could contribute to improve the risk of HF and the cardiovascular profile of these patients (88).

Renal benefits were observed in the AMPLITUDE-O Trial. This study evaluates the effects of efpeglenatide on cardiovascular and renal outcomes in T2DM patients at risk of CV events. The rate of cardiovascular and renal outcomes was defined as decreased renal function or macroalbuminuria, and was lower in the efpeglenatide group. The AWARD-7 trial (Dulaglutide versus insulin glargine in patients with type 2 diabetes and CKD) was the first RCT to examine the efficacy of GLP-1 RA (dulaglutide) in moderate to severe CKD. Mean eGFR was 38 m/min/1.73 m^2^, with a mean decrease per year of 3.3 ml/min/1.73 m^2^ in patients treated with insulin versus −0.7 ml/min/1.73 m^2^ with dulaglutide. In addition, among patients at increased risk of renal disease progression (macroalbuminuria; UACR >300 mg/g), the attenuation of the mean decline in eGFR was maintained, and, in the high-dose dulaglutide group, fewer patients achieved the combined endpoint of ESKD or >40% deterioration of GFR, versus the insulin group (5.2% vs. 10.8%; *p* = 0.038). The LEADER (*liraglutide* vs. *placebo*) and SUSTAIN-6 (*semaglutide* vs. *placebo*) trials showed a lower number of cardiovascular events and a lower risk of development and progression of CKD, as a result of a reduction in the appearance of macroalbuminuria. In this sense, in a meta-analysis that involved >56,000 patients with DM2 treated with GLP-1 RA, beneficial cardiovascular, renal, and mortality effects were found (89). The FLOW trial (*Semaglutide Compared to Placebo in People With Type 2 Diabetes and Chronic Kidney Disease; URL:* ClinicalTrials.gov*. Unique identifier: NCT03819153*) will be the first large RCT with DM2 and CKD patients, treated with semaglutide versus placebo, to evaluate primary renal events.

## 6. Pipeline treatment for CRS in diabetes

Although prevention of cardiorenal complications in patients with diabetes has improved with drug classes like RASi, SGLT2i or GLP-1 RAs, there is a risk of CKD progression and cardiovascular events in patients with diabetes (90). In the CREDENCE (100% of patients with T2DM), DAPA-CKD (67.5% of patients with T2DM), and EMPA-KIDNEY (44.4% of patients with T2DM), the groups of patients that received the SGLT2i on top of standard care still showed a risk of 9%–13% of developing renal events after a median follow-up of 2.5 years (64–66). Furthermore, all-cause mortality risk was 5%–7% in these groups. Thus, there is still a pressing need to develop new therapeutic agents that prevent both CV and renal events in these patients.

Mineralocorticoid receptor blockade is among the promising new add-on therapies to the current standard care. Classical mineralocorticoid receptor antagonists (MRAs), spironolactone and eplerenone, already showed a reduction in CV events or hospitalization due to HF in studies that included 30%–35% of patients with diabetes without advanced DKD (91). Despite this, classical MRAs have not been studied with prespecified renal outcomes and with the inclusion of patients with overt DKD in large randomized clinical trials. In addition, the probability of hyperkalemia and acute kidney injury is increased when RASi are added, limiting the use of this class of drugs in the T2DM patient (92). Recently, the non-steroidal MRAs such as finerenone have shown effects as potent as spironolactone and a higher selectivity when compared to eplerenone. The FIDELITY study combined the results of the FIDELIO-DKD (Finerenone in Reducing Kidney Failure and Disease Progression in Diabetic Kidney Disease) (93) and FIGARO-DKD (94) RCT. In the pooled analysis of 13,026 patients treated with finerenone or placebo and followed for a median of 3 years, there was evidence of a 23% reduction in the relative risk of developing a sustained decline of ≥57% from baseline in GFR or death due to renal causes (95). Additionally, finerenone provided a 14% reduction in the relative risk of death from cardiovascular causes, nonfatal myocardial infarction, nonfatal stroke, or hospitalization for HF, and the improvement in the composite cardiovascular outcome was due largely to a decrease in HF hospitalizations. However, there was a clear trend to reduce death secondary to CV causes (HR 0.88 (95% CI: 0.76‒1.02, *p* = 0.092), that would be more evident if the follow-up times had been longer (95). It is worth mentioning that the FIDELITY analysis also included 6.7% of patients previously treated with SGLT2i, and showed a similar number of CV and renal events regardless the previous use of SGLT2i. Because both drug classes have different mechanisms of action and SGLT2i could prevent hyperkalaemia secondary to finerenone use, it is expected to see a synergistic protection with the combination. However, studies that include a larger number of patients treated with both SGLT2i and finerenone are needed.

Obesity is an important cause of DM and increased cardiovascular risk worldwide (96). An increase in 1 standard deviation of the BMI was related to 1.6-fold higher risk of presenting diabetes (97). In the same line, weight loss has been related to a reduction in albuminuria as well as renal and CV events. In fact, the cardiorenal benefits of previously mentioned SGLT2i and GLP-1 RAs can be partly ascribed to weight loss. Recently, a dual glucose-dependent insulinotropic polypeptide-GLP-1 RA with long half-life has been evaluated in RCT against a semaglutide 1 mg showing a 0.5% greater reduction in glycated hemoglobin. High doses of tirzepatide (15 mg) also obtained almost 5 kg more body weight reduction compared to semaglutide after 40 weeks of treatment. Thus, the dual agonism is clearly superior in reducing body weight when compared to single GLP-1 RA. The central glucose-dependent insulinotropic polypeptide receptor effect of tirzepatide adds to the GLP-1 AR, allowing a more substantial reduction in food intake, leading to a further drop in body weight (98, 99). In addition, we have some promising and recent *post-hoc* analysis that show a reduction in albuminuria and renal events when comparing tirzepatide versus insulin glargine in 1.6 years follow-up (100). In this line, further RCT will show if tirzepatide has greater or similar long-term cardiorenal protective effects when compared to single GLP-1 RAs already approved.

Another drug class, that has already been evaluated in RCT due to its renal protective effects, is the selective endothelin receptor antagonists (ERAs). Endothelin receptor antagonists prevent endothelin's deleterious effects, being endothelin receptor A (ET_A_) the receptor most frequently involved in the negative effects of the endothelin pathway (vasoconstriction, endothelial injury, or podocyte injury) (101). Thus, endothelin receptor blockade has been traditionally directed to ET_A_ blockade. ET_A_ antagonism has shown renal protective effects, but fluid overload caused by the blockade of the ET_A_-mediated natriuretic effects has been an important limitation. In 2009 the ASCEND trial that included T2DM patients that were treated with avosentan (ET_A:B_ selectivity 50–300:1) was stopped prematurely after a higher incidence of fluid overload and HF events in the active-treatment arm (102). Nevertheless, avosentan showed a 30% median reduction in albuminuria compared to placebo. In order to avoid fluid retention, a more recent trial with atrasentan (ET_A:B_ selectivity 1,200:1) randomized only responder patients (reduction of 30% in albuminuria without weight gain or increase in BNP) to receive active treatment or placebo. The atrasentan group showed a reduction in renal events and HF was similar between the groups, after 2.2 years of follow-up (103). However, in the ET_A_ antagonist group, fluid retention and anemia were more frequent. Recently, it has been hypothesized that the combination of SGLT2i and ERAs could have synergistic cardiorenal protective effects, as both drug classes act through different pathways, and SGLT2i could prevent the fluid overload and anemia linked to the use of ERAs (104). In fact, preclinical studies in T2DM mice have shown enhanced cardiorenal protective effects (105). Ongoing trials like ZENITH-CKD will clarify the future use of ERAs in patients with CKD and may give some new insights about their use in cardiorenal syndrome.

The soluble guanylate cyclase (sGC) stimulator is a drug class more recently studied in RCT. Endothelial nitric oxide (NO) has cardiovascular protective effects, including vasodilation, reduction of vascular oxidative stress, improved diastolic relaxation, and inhibition of fibrosis and smooth muscle cell proliferation. The downstream effects of the NO pathway are mediated by sGC, which converts guanosine triphosphate (GTP) into cyclic guanosine monophosphate (cGMP) and further activates intracellular cGMP-dependent intracellular protein kinases. Thus, sGC stimulators enhance the latter pathway and promote cardiovascular protective effects. A RCT evaluating treatment with vericiguat (an oral sGC stimulator) in patients with HFrEF that included 46.9% patients with diabetes showed a reduction of the primary CV composite outcome after a median follow-up of almost 11 months (106). The reduction was mainly due to a decrease in hospitalization for HF. Moreover, a non-significant reduction of death due to CV causes was observed that could have been evident if follow-up times had been longer.

Additionally, several new drugs classes are being evaluated in preclinical studies. The apelin pathway may have promising effects in the treatment of CRS in patients with diabetes as it improves inotropism, produces vasodilation, and has aquaretic effects (107). These effects are in part ascribed to the increased production of endothelial NO, and results in animal studies have been promising (108). Moreover, apelin pathway stimulation may exert protection against kidney fibrosis and podocyte loss in diabetes. Other drug classes with renal protective effects are the hypoxia-inducible factor (HIF) prolyl hydroxylase inhibitor or the advanced glycation end product (AGE) (109). The AGE inhibitors have shown controversial results in DKD, but preclinical studies evaluating HIF prolyl hydroxylase inhibitors have displayed protection against renal tubular injury that can be of interest in patients with diabetes.

## 7. Conclusion

The triad of CKD, DM, and HF frequently go hand in hand, and the presence of all three entails an increased risk of hospitalization, as well as mortality. Understanding the pathophysiology of this interaction is essential for an early diagnosis of these patients, which allows us to offer them the best treatment. The appearance of new drugs, such as SGLT2i, GLP-1 RA represents a substantial improvement for these cardiorenal patients, where until now we had little to offer. Other drugs, such as non-steroidal MRAs and tirzepatide, have shown promising results that will change, in the short term, the management of renal and cardiorenal patients. The complexity of these patients calls for a multidisciplinary approach for better results.
